# Overexpression of UbcH10 alternates the cell cycle profile and accelerate the tumor proliferation in colon cancer

**DOI:** 10.1186/1471-2407-9-87

**Published:** 2009-03-21

**Authors:** Takeo Fujita, Hirokuni Ikeda, Naruto Taira, Shinji Hatoh, Minoru Naito, Hiroyoshi Doihara

**Affiliations:** 1 Department of Cancer and Thoracic Surgery, Okayama University School of Medicine, Okayama, Japan; 2 Division of Breast and Endocrine Surgery, Okayama University Hospital, Okayama, Japan; 3 Division of Gastrointestinal Surgery, Okayama University Hospital, Okayama, Japan

## Abstract

**Background:**

UbcH10 participates in proper metaphase to anaphase transition, and abrogation of UbcH10 results in the premature separation of sister chromatids. To assess the potential role of UbcH10 in colon cancer progression, we analyzed the clinicopathological relevance of UbcH10 in colon cancer.

**Methods:**

We firstly screened the expression profile of UbcH10 in various types of cancer tissues as well as cell lines. Thereafter, using the colon cancer cells line, we manipulated the expression of UbcH10 and evaluated the cell cycle profile and cellular proliferations. Furthermore, the clinicopathological significance of UbcH10 was immunohistologically evaluated in patients with colon cancer. Statistical analysis was performed using the student's t-test and Chi-square test.

**Results:**

Using the colon cancer cells, depletion of UbcH10 resulted in suppression of cellular growth whereas overexpression of UbcH10 promoted the cellular growth and oncogenic cellular growth. Mitotic population was markedly alternated by the manipulation of UbcH10 expression. Immunohistochemical analysis indicated that UbcH10 was significantly higher in colon cancer tissue compared with normal colon epithelia. Furthermore, the clinicopathological evaluation revealed that UbcH10 was associated with high-grade histological tumors.

**Conclusion:**

The results show the clinicopathological significance of UbcH10 in the progression of colon cancer. Thus UbcH10 may act as a novel biomarker in patients with colon cancer.

## Background

Proper cell cycle progression is orchestrated by the controlled oscillation of a series of cell cycle events. Deregulation of appropriate cell cycle control often results in chromosomal instability, which is a potential trigger for the initiation of cancer [1]. Dozens of molecules are expressed and degraded in specific phases of the cell cycle through the ubiquitin/proteasome pathway, and the ubiquitin/proteasome system has been linked to the orchestration of several important cell cycle events, such as proteolysis of cyclin-dependent kinase and their inhibitors [2-4]. In this system, substrate molecules are regulated for degradation by ubiquitin activating enzyme (E1), ubiquitin conjugating enzyme (E2), and E3 ligase. Of these, two major E3 ligases control a critical cell cycle stage. APC (anaphase-promoting complex) mainly plays a role in the transition from mitosis to G1, whereas the SCF (Skp1-cullin-F box) complex plays a critical role from G1 to S [5,6]. During the transition from mitosis to G1, APC activity is mainly regulated by the coactivators Cdc20 and Cdh1. As cells enter mitosis, Cdc2 kinase enhances the formation of active Cdc20/APC complex, which induces securin degradation. This, in turn, induces the separation of sister chromatids through the activation of separase. In late mitosis, Cdc20 is degraded by Cdh1/APC and leads to the complete replacement of Cdc20/APC by Cdh1/APC [7,8]. Recent studies demonstrated that UbcH10 supplementation promotes dissociation of the spindle assembly checkpoint proteins Mad2 and BubR1 from Cdc20, and then activates Cdc20/APC, which leads to cyclin A and securin degradation [9,10]. These results suggest that UbcH10 is potentially involved in the termination of the spindle assembly checkpoint and further implies that aberrant UbcH10 expression impairs the spindle assembly checkpoint resulting in chromosomal instability [11,12].

Previous epigenetic studies using a wide variety of cancers have demonstrated that molecules that are associated with the spindle assembly checkpoint aberrantly express in certain malignancies [13-15]. Indeed, dysfunction of several components of the spindle assembly checkpoint including Mad1, Mad2, BubR1, and Aurora A are correlated with chromosomal instability in malignant tumors [13-15]. Moreover, some of these molecules are involved in determining the efficacy of specific chemotherapeutic agents [16,17]. Therefore, an investigation of spindle checkpoint molecules will enhance our molecular background knowledge and lead us towards a potential treatment for cancers.

Previous work with a cell line-based assay has demonstrated UbcH10 involvement in chromosomal instability [18-20]. To further validate the connection between UbcH10 status and colon cancer progression, we developed a cell-line based assay and tissue array analyses to elucidate the clinicopathological relevance of UbcH10 in colon cancer. The results confirmed that aberrant UbcH10 expression promotes tumor formation by deregulating the normal progression of the cell cycle. Thus, these results provide evidence for the involvement of the spindle assembly checkpoint in cancer and may possibly encourage the exploration of the cell cycle checkpoint machinery associated with clinical oncology.

## Methods

### Plasmid preparation and small interfering RNA

The preparation of pcDNA3-Flag and pcDNA3-Flag-UbcH10 plasmids have been previously reported [18]. Knockdown using small interfering RNA (siRNA) for UbcH10 was carried out using the following target sequence: (UbcH10 495- 5'-AACCTGCAAGAAACCTACTCA-3') [19]. Transfection was conducted using Lipofectamine2000 (Invitrogen, Carlsbad, CA) according to the manufacturer's protocol. Thereafter, cells were cultured McCoy's medium (GIBCO, Carlsbad, CA) supplemented with 10% FBS and 1% penicillin/streptomycin solution (GIBCO, Carlsbad, CA) for 10–14 days in the presence of 500 μg/ml of G418 (Promega, Madison, WI) and positive clones were selected in the presence of G418 (Promega, Madison, WI).

### Antibodies and reagents

Antibodies against UbcH10 were purchased from Boston Biochem (Cambridge, MA), and tubulin was purchased from Calbiochem (Gibbstown, NJ). Western blot analysis was performed using the ECL detection kit (Amersham, Buckinghamshire, UK).

### Cell cycle analysis

Cell cycle analysis was carried out by propidium iodide (Sigma-Aldrich, St. Louis, MO) staining and fluorescent activated cell sorting (FACS) analysis. Flow cytometric analysis of the stained cells was performed using a FACScan (Becton Dickinson, Mountain View, CA).

### Colony formation by soft agar assay

After transfection, viable cells were counted (2.0 × 10^5^/mL) and seeded onto soft agar [21] with slight modification (Dr. Erik Flemington, Tulane Cancer Center, New Orleans, LA). Briefly, a 1% agarose solution was prepared with sterile water, the agarose was pipetted into each well to make a thin film, and the cells were plated. Seven days after seeding, colony formation was assessed by examination and counting under a microscope. Because the aggregates of the untreated cells did not grow during the experimental period, they were not considered colonies. Each experiment was repeated at least thrice and the values are given as the results of mean (± S.D) value score.

### Immunofluorescence

Immunofluorescence analysis was performed using the phosphorylated histone H3 (Cell Signaling, Boston, MA) as the primary antibody and fluorescein isothiocyanate (FITC) as the secondary antibody (Jackson ImmunoResearch, West Grove, PA). The experiment was repeated at least three times.

### Immunohistochemical staining and prognostic analysis

Samples were deparaffinized, rehydrated, and the antigen was retrieved in citrate buffer. Then the sections were treated with hydrogen peroxide. Samples were incubated using the UbcH10 antibody followed by the secondary antibody (Vector Laboratories, Burlingame, CA), and were then incubated with avidin-biotin peroxidase complex solution (DAKO Cytomation, Carpinteria, CA) and 3-amino-9-ethylcarbazole solution (DAKO Cytomation, Carpinteria, CA). Tissue arrays were purchased from US Biomax. (Rockville, MD). All patients provided the written informed consent before analyze the surgically removed materials. The expression of each molecule was tested in cancer and normal-matched adjacent tissues. The specificity and optimal concentration of the antibody was verified using the test tissue array slide.

### Scoring of immunohistochemical staining

Staining intensity and subcellular localization were evaluated twice in a blinded manner based on a pre-agreed staining scoring standard obtained from an expert pathologist (Dr. Cheng, Department of Pathology, University of Pittsburgh). Staining intensity was scored using the following criteria: (*a*) 0–1, negative or low staining intensity in >50% of the tumor cells or moderate to high in <50% of the cells (low) and (*b*) 2–3, moderate to high staining intensity in >50% of tumor cells [22].

### Statistical analysis

Each value represents at least three independent experiments. Statistical significance was evaluated with the two-tailed Student's *t*-test and the Chi-square test. Fisher's exact test was used for the analysis of the immunostaining results and the clinicopathological data. *p *< 0.05 was considered statistically significant. All data were analyzed with SPSS 14.0 (Chicago, IL) for Windows.

## Results

### Higher levels of UbcH10 in human cancer

**Figure 1:**
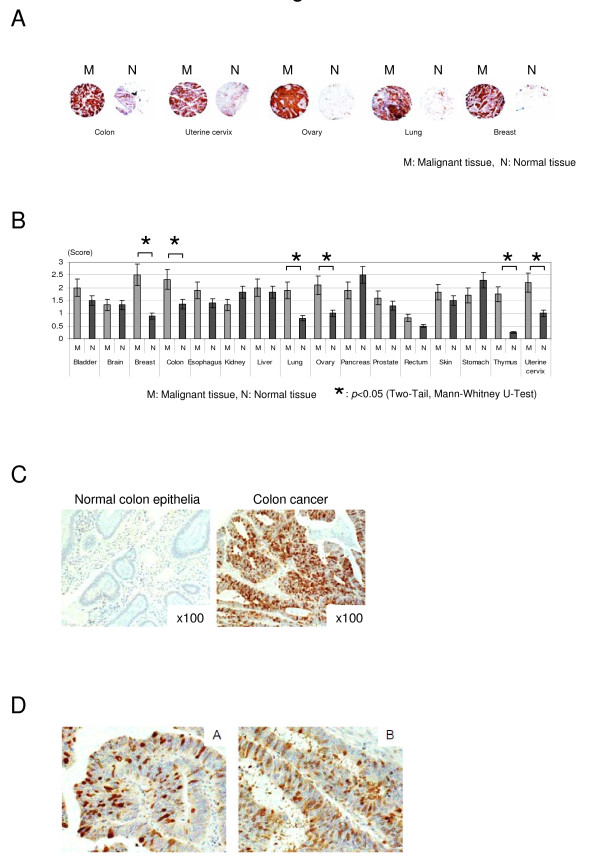
**Aberrant expression of UbcH10 in cancer**. (A) Immunohistochemical analysis of the UbcH10-expression profile in various types of cancer including 16 different organs revealed that UbcH10 is highly expressed in cancer compared with its adjacent non-malignant tissues of the colon, breast, lung, ovary, thymus, and uterine cervix. (B) Summary of the UbcH10-expression profile in human tissues. Statistically significant differences were observed in the UbcH10 level between cancer and normal adjacent tissues. (C) Representative picture of the immunohistochemical analysis using normal colon epithelia and colon cancer tissue. UbcH10 is markedly expressed in colon cancer compared with normal colon epithelia. (D) Results of the immunostaining with UbcH10 in colon cancer. Higher levels of UbcH10 appeared in cancer, and it was mainly localized to the nucleus.

It has been demonstrated that appropriate mitotic regulation is essential for the proper progression of the cell cycle; else abrogation of mitotic machinery might result in tetraploidy or aneuploidy [8,9]. UbcH10 is thought as an oncogenic potential enhancing carcinogenesis, according to several large genome wide studies as well as cell line based biophysical analysis. Previous biochemical studies have demonstrated that UbcH10 plays a pivotal role in orchestrating the metaphase to anaphase transition via ubiquitin-proteosome pathway [10,20,23], supporting the results of prior epigenetic studies. We analyzed UbcH10 expression in various types of cancers to further validate the oncogenic potential of UbcH10 in malignant tumors status. Using a tissue array, we scored the staining intensity of more than 500 tissue samples that had 16 types of cancer together with non-malignant tissues. UbcH10 was highly expressed in several cancer types compared with the adjacent non-malignant area (Fig. 1A). Staining was significantly more intense between breast, colon, lung, ovary, and cervical cancer tissues than in non-malignant tissue (Fig. 1B, C), and UbcH10 was most predominantly seen in the nucleus but could also be found in the cytoplasm of colon cancer cells (Fig. 1D). There was no significant difference in UbcH10 cellular localization between colon cancer and normal colon epithelial tissues (data was not shown). Taken together, based on immonohistochemical analysis, these results imply that impairment of cell cycle regulation by aberrant UbcH10 may be related with colon cancer.

### Overexpression of UbcH10 enhances cellular proliferation in colon cancer cells

**Figure 2:**
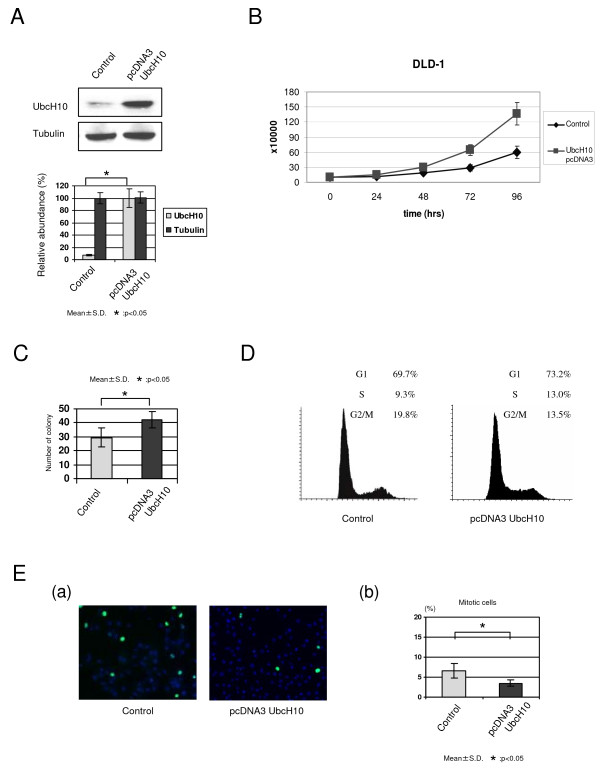
**Overexpression of UbcH10 promotes the cellular proliferation and alters the cell cycle profile in colon cancer**. (A) UbcH10 overexpression in DLD1 colon cancer cells. (B) Cellular proliferation was markedly increased in UbcH10-overexpressed DLD1 cells compared with control cells. (C) Results of anchorage-independent colony formation soft-agar assay. The number of colonies was markedly increased in stably-transfected UbcH10 DLD1 colon cancer cells. (D) Cell cycle profile of overexpresed UbcH10 and control DLD1 cells shows that the population of G2/M cells is moderately decreased with the delivery of UbcH10. (E) a. Immunofluorescence with phosphorylated-histone H3 (pH3). The population of pH3 positive cells was decreased in UbcH10-overexpressed DLD1 cells. b. Quantification of immunofluorescence analysis (triplicate experiments).

To analyze the role of UbcH10 in promoting cellular growth associated with cell cycle progression through mitosis, we overexpressed UbcH10 in colon cancer cells (Fig. 2A). As shown in Fig. 2B, UbcH10 overexpression in DLD1 cells resulted in a significant acceleration of cellular growth. There are remarkable changes of doubling times by the overexpression of UbcH10. Calculated doubling times of DLD1 cells; 37.0 hrs (control cells), 21.5 hrs (UbcH10-overexpressed cells), respectively. To test the effect of UbcH10 on cell growth and oncogenic colony formation, we conducted an anchorage-independent growth assay and further measured the cell cycle profile in DLD1 cells. UbcH10 overexpression promoted an increase in the number and size (data not shown) of colonies on soft agar (Fig. 2C). Recent studies have demonstrated that UbcH10 promotes the metaphase to anaphase transition. To validate the role of UbcH10 in cell cycle regulation, we examined the cell cycle profile in UbcH10 overexpressed colon cancer cells. UbcH10 overexpression in DLD1 cells reduced the G2/M-cell fraction (Fig. 2D). To determine whether a decrease in the G2/M fraction was dependant on the G2 or M phase, we analyzed the mitotic population with immunofluorescence using phosphorylated-histone H3. As shown in Fig. 2E, the percentage of mitotic cells was markedly reduced in UbcH10-abundant DLD1 cells compared with control cells. These results suggest that increases of UbcH10 protein levels could lead to aberrant cell cycle progression, particularly during mitosis, which, in turn, could promote oncogenesis in colon cancer cells.

### UbcH10 depletion suppresses colon cancer cell proliferation

**Figure 3:**
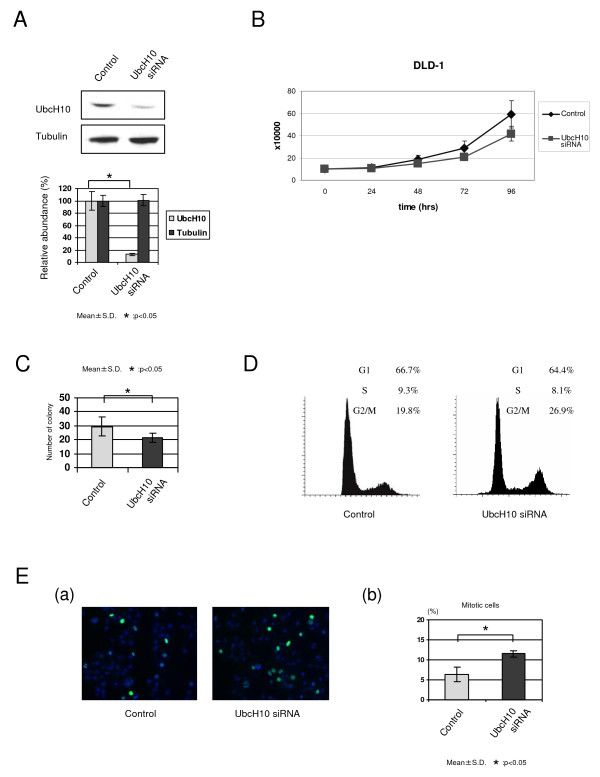
**Depletion of UbcH10 suppresses cellular proliferation in breast cancer cells**. (A) UbcH10 knockdown in DLD1 colon cancer cells. (B) The cellular proliferation rate was decreased in DLD1 cells by targeting siRNA for UbcH10. (C) Results of the anchorage independent colony formation assay using soft agar. The number of colonies was markedly decreased in DLD1 colon cancer cells by the delivery of UbcH10-siRNA. (D) Cell cycle patterns of UbcH10-knockdown and control DLD1 cells revealed that populations of G2/M cells increase with UbcH10depletion. (E) a. Results of the immunocytochemical analysis using phosphorylated-histone H3 in DLD1 cells. UbcH10 knockdown increased the population of pH3 positive cells. b. Quantification of mitotic cells (triplicate experiments).

We conducted UbcH10 depletion studies in colon cancer cells (Fig. 3A) and examined the effects on cellular proliferation as well as colony formation. As shown in Fig. 3B, UbcH10 knockdown largely reduced cellular proliferation in DLD1 cells. There are remarkable changes of doubling times by the depletion of UbcH10. Calculated doubling times of DLD1 cells; 37.0 hrs (control cells), and 53.7 hrs (UbcH10-depleted cells), respectively. Control DLD1 cells showed a high capacity for colony formation in soft agar, whereas both cell number and colony size decreased significantly when UbcH10 was depleted. This result confirms a potential oncogenic effect of UbcH10 in colon cancer cells (Fig. 3C). The G2/M cell fraction was significantly elevated in UbcH10-depleted DLD1 cells (Fig. 3D). Consistent with the results of the above experiment, there were an increased number of cells positive for phosphorylated-histone H3 in the absence of UbcH10 (Fig. 3E). These results further suggest that UbcH10 promotes oncogenic proliferation and accelerates tumor growth, possibly through mitotic progression.

### Clinicopathological relevance of UbcH10 in patients with colon cancer

**Figure 4:**
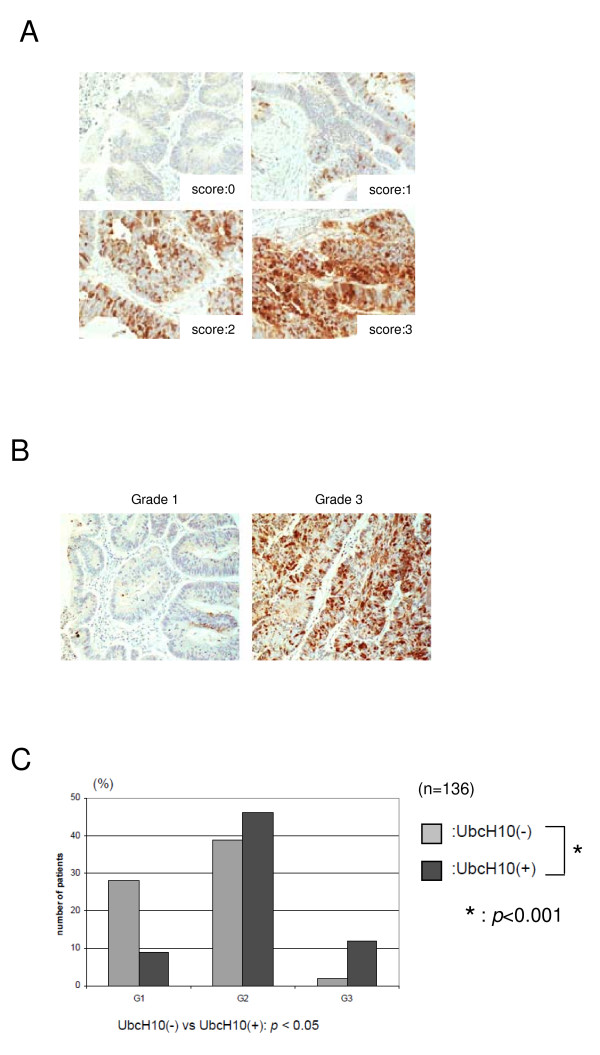
**The clinicopathological significance of UbcH10 in patients with colon cancer**. (A) Representative picture of negatively or positively stained colon tissue samples. (B) Representative picture of breast cancer tissues immunohistochemically tested for UbcH10. UbcH10 was more abundantly expressed in grade 3 tumors compared with grade 1 tumors. There was no remarkable difference in UbcH10 cellular localization with regard to histological grade, whereas UbcH10 was relatively highly detected in the nucleus. (C) Summary of the level of UbcH10 and histological grade (G1-3) in patients with colon cancer.

A potential function of UbcH10 in promoting progression of colon cancer via regulation of mitotic checkpoint can be revealed from the previous experiment. To correlate our in vitro results to clinical or pathological relevance, we evaluated the clinicopathological significance of UbcH10 in patients with colon cancer. In this study, we used an independent set of 150 colon-neoplasm samples that included four different types of normal colon epithelium as controls. We analyzed the patient age, gender, tumor size, lymph node status (N0-2), histological grade (G1-3), and histological type of tumor and evaluated the clinicopathological relevance of UbcH10 in these patients. Among the 150 patients, 69 patients (46.0%) were histologically positive for UbcH10 and none of the controls were UbcH10 positive. This result is consistent with our previous observation (Fig. 1). There were no significant differences between UbcH10 positive and negative cancers with respect to patient age (p = 0.674), gender (p = 0.779), tumor size (p = 0.601), lymph node metastasis (p = 0.587), or histological type of cancer (p = 0.174) (Table 1). However, UbcH10 positive cancer was more frequently and significantly categorized as a high-grade histological tumor [Grade 3; UbcH10 (-): 2.98%, UbcH10 (+): 17.91%] (p < 0.001; Fig 4A–C). Results of this analysis indicate that UbcH10 abundance is correlated with high-grade histological tumors and suggests that higher levels of UbcH10 could be associated with aggressive cellular behavior and a potentially poor prognosis in patients with colon cancer. Taken together, these results show that UbcH10 has a substantial function in promoting colon cancer progression and could be a potential prognostic marker in patients with colon cancer.

**Table 1:** Differences between UbcH10 positive and negative cancers.

	UbcH10(-)	UbcH10(+)	P-value
***Age***			
65≥	48	45	0.674
65>	22	24	
			
***Gender***			
F	42	43	0.779
M	28	26	
			
***Tumor size***			
T1.2	29	26	0.601
T3,4	40	43	
			
***Lymph node***			
N0	52	45	0.587
N1	19	23	
N2	0	2	
			
***Histological Grade***			
G1	28	9	<0.001
G2	39	46	
G3	2	12	
			
***Histological type***			
Adenocarcinoma	67	69	0.174
Mucinous Carcinoma	2	0	
Signet ring-cell carcinoma	2	0	
Adenoma	6	0	

## Discussion

Appropriate cell cycle progression is orchestrated by a series of molecular events in which the ubiquitin/proteasome system is strongly associated with the cell cycle oscillation machinery [1,2]. Therefore, dysfunction of this system often leads to chromosomal instability and eventually results in the initiation of diseases, such as malignant tumors [5,6]. The ubiquitin/proteasome system includes ubiquitin activating enzyme (E1), ubiquitin conjugating enzyme (E2), and E3 ligase. Two major E3 ligases, SCF and APC, are critical for governing mitosis or G1/S progression [5-7]. Loss of function of the SCF or APC pathways is involved in the initiation or progression of human cancer [24-26]. Previous work also provides evidence that E2 proteins play an important role in regulating cell cycle progression. Indeed, recent studies demonstrate that UbcH10 promotes the dissociation of Mad2 from Cdc20, a crucial step in the metaphase to anaphase transition in which important molecules are involved in the organization of appropriate spindle assembly checkpoints [9,10]. The function of the spindle assembly checkpoint is to ensure the proper separation of duplicated daughter genomes during mitosis, and the dysfunction of this system often results in aneuploidy or tetraploidy [25,26]. In fact, abnormal levels of Mad1, Mad2, BubR1, and aurora A are observed in prostate, stomach, and lung cancers [13-15,27]. Our results using cultured cells, as well as the epigenetic analysis, further confirms that dysfunction of the spindle assembly checkpoint could potentially induce the initiation or progression of cancer.

Abnormal levels of UbcH10 promote aberrant cell cycle progression and are potentially associated with tumor progression [18-20,27]. Cell culture studies have indicated the potential oncogenic role of UbcH10 [18,27]. Given the correlation between mitotic machinery dysfunction and chromosomal instability, the association between UbcH10 and mitotic regulation further implicates the involvement of UbcH10 in tumorigenesis [18,20,27]. Our results suggest that abnormal levels of UbcH10 increase oncogenic potential and accelerate cellular proliferation. Furthermore, UbcH10 overexpression or knockdown induced significant changes in the cell cycle profile and the properties of oncogenic growth in colon cancer cells, which is consistent with the prior observation that UbcH10 participates in the progression of cancer.

### Clinicopathological analysis confirms the oncogenic role of UbcH10

The results of our examination of the association between UbcH10, lymph-node metastasis, and histological grade of colon cancers provoked the hypothesis that UbcH10 could promote tumor growth via abrogation of the spindle assembly checkpoint [18,27]. Our analysis indicated that UbcH10-negative colon cancers were associated with a low histological grade and the loss of aggressive cancer behavior. Thus, this potentially links UbcH10 activity to the biological characteristics of tumor. Indeed, previous results of large-scale genetic screening studies have revealed that UbcH10 is one of the candidate molecules related to aggressive behavior of the tumors [28-33]. Therefore, our clinicopathological assessment of UbcH10 is compatible with prior epigenetic and biological studies that have implicated UbcH10 as a predictor of the biological characteristics of cancer. Furthermore, lower levels of p31comet, another molecule that induces the metaphase to anaphase transition, also acts as a potential prognostic marker in cancer [34,35]. Moreover, Usp44 inhibits Cdc20 degradation and counteracts UbcH10 to decelerate the metaphase to anaphase transition [36]. Therefore, a balance between UbcH10 and Usp44 could determine the appropriate timing of sister chromatid separation and further explain the significance of UbcH10 in cancer [36].

Our study had limitation. We could not adequately address the phenomenon that overexpression of UbcH10 induced cellular proliferation while contradictory decreased the population of mitosis. However, our results were consistent with the results of previous literatures, which may even be considered as strength of the present study. Currently, role of UbcH10 is suggested to be only at the end of G1-phase, being inconsistent with both in the spindle checkpoint and inactivation of the APC/C [37]. Their suggestion would be the potential clue to explain the contradictory phenomenon, and further investigation is required to unveil the controversial point of UbcH10.

## Conclusion

Dysfunction of the ubiquitin/proteasome system has been strongly linked to carcinogenesis through its disruption of the balance between oncoproteins and tumor suppressor proteins. Disruption of mitotic regulation at key sites during the cell cycle can lead to genomic instability and uncontrolled growth. The spindle assembly checkpoint is a critical point that dictates chromatids separation as well as the orchestration of appropriate cellular proliferation. Our analyses verified the importance of the ubiquitylation regulatory cascade in which one E2 protein participates in the determination of mitotic progression. The UbcH10-expression pattern in cancer supports the notion that this regulatory axis controls cellular proliferation and that its abrogation leads to carcinogenesis. These results provide a further understanding of UbcH10 and its role in cell cycle regulation and colon cancer formation.

## Competing interests

The authors declare that they have no competing interests.

## Authors' contributions

TK participated in the draft manuscript. TK and IH participated in the design of the study, carried out the molecular and histological study. NT, HI, MN, HD participated in the design of the study. All authors read and approved the final version of the manuscript.

## Pre-publication history

The pre-publication history for this paper can be accessed here:

http://www.biomedcentral.com/1471-2407/9/87/prepub
